# Bacterial Chromosome Replication and DNA Repair During the Stringent Response

**DOI:** 10.3389/fmicb.2020.582113

**Published:** 2020-08-28

**Authors:** Anurag Kumar Sinha, Anders Løbner-Olesen, Leise Riber

**Affiliations:** Department of Biology, University of Copenhagen, Copenhagen, Denmark

**Keywords:** (p)ppGpp, DNA replication, DNA repair, stringent response, genome stability, *Escherichia coli*, *Bacillus subtilis*

## Abstract

The stringent response regulates bacterial growth rate and is important for cell survival under changing environmental conditions. The effect of the stringent response is pleiotropic, affecting almost all biological processes in the cell including transcriptional downregulation of genes involved in stable RNA synthesis, DNA replication, and metabolic pathways, as well as the upregulation of stress-related genes. In this Review, we discuss how the stringent response affects chromosome replication and DNA repair activities in bacteria. Importantly, we address how accumulation of (p)ppGpp during the stringent response shuts down chromosome replication using highly different strategies in the evolutionary distant Gram-negative *Escherichia coli* and Gram-positive *Bacillus subtilis.* Interestingly, (p)ppGpp-mediated replication inhibition occurs downstream of the origin in *B. subtilis*, whereas replication inhibition in *E. coli* takes place at the initiation level, suggesting that stringent cell cycle arrest acts at different phases of the replication cycle between *E. coli* and *B. subtilis*. Furthermore, we address the role of (p)ppGpp in facilitating DNA repair activities and cell survival during exposure to UV and other DNA damaging agents. In particular, (p)ppGpp seems to stimulate the efficiency of nucleotide excision repair (NER)-dependent repair of DNA lesions. Finally, we discuss whether (p)ppGpp-mediated cell survival during DNA damage is related to the ability of (p)ppGpp accumulation to inhibit chromosome replication.

## Introduction

Bacteria respond to a variety of changing environmental conditions by inducing the stringent response. Known inducers of the stringent response include nutrient limitations such as amino acids, fatty acids, carbon and nitrogen starvation, and other stresses such as high temperature and low pH (Gallant et al., 1977; Gentry and Cashel, 1996; Wells and Gaynor, 2006; Winther et al., 2018; Sinha et al., 2019; Schafer et al., 2020). The hallmark of stringent response is the accumulation of guanosine tetra- or pentaphosphate, ppGpp and pppGpp, respectively [collectively called (p)ppGpp or alarmone], which leads to reprogramming of cell physiology facilitating cell survival under stress (Potrykus and Cashel, 2008; Hauryliuk et al., 2015). Importantly, (p)ppGpp plays a role in antibiotic tolerance and is essential for virulence in pathogenic bacteria (Dalebroux et al., 2010; Hauryliuk et al., 2015). Additionally, (p)ppGpp regulates bacterial growth rates even in the absence of external environmental stress (Potrykus et al., 2011).

Alarmones are synthesized and hydrolyzed by the long RelA/SpoT Homolog (RSH) protein superfamily. In the Gram-negative γ–proteobacterium *Escherichia coli*, two paralogous enzymes modulate (p)ppGpp levels; monofunctional RelA, which has only synthetase activity, and bifunctional SpoT, which has both synthetase and hydrolase activities. In the spore-forming Gram-positive bacterium, *Bacillus subtilis*, (p)ppGpp levels are metabolized by one long RSH superfamily protein Rel and two small alarmone synthetases (SASs) called RelP and RelQ (Liu et al., 2015; reviewed in Ronneau and Hallez, 2019). Accumulation of (p)ppGpp rapidly alters the levels of a wide range of gene transcripts and metabolites to allow cell survival and adaptation to new growth conditions (Eymann et al., 2002; Traxler et al., 2008). The major changes involve transcriptional down-regulation of genes involved in stable RNA (rRNA and tRNA) synthesis, DNA replication, and metabolic pathways, whereas genes engaged in stress and amino-acid biosynthesis are activated (Sanchez-Vazquez et al., 2019; Gummesson et al., 2020). In *E. coli*, (p)ppGpp directly binds two sites on RNA polymerase (RNAP) to allosterically alter its binding to- and efficiency at different gene promoters, which results in genome-wide transcriptional reprogramming. (p)ppGpp binding to RNAP and the consequent RNAP-driven transcriptional response is potentiated by another small RNAP binding protein, DksA (reviewed in Gourse et al., 2018). In *B. subtilis*, RNAP lacks critical (p)ppGpp binding sites and no DskA homologs have been identified. As a consequence, (p)ppGpp does not directly target *B. subtilis* RNAP. Instead (p)ppGpp synthesis strongly depletes the pool of available GTP, which leads to an indirect inhibition of stable RNA promoter activity since GTP is used as start nucleotide for most of the stable RNAs (Krasny and Gourse, 2004; Gourse et al., 2018; Sanchez-Vazquez et al., 2019). Importantly, apart from transcriptional responses, (p)ppGpp directly targets many other proteins to affect metabolic processes such as nucleotide metabolism and biosynthetic pathways (Zhang et al., 2018, 2019; Wang et al., 2019).

Here, we discuss how the stringent response affects chromosome replication, DNA damage and repair activities, focusing mainly on recent studies done in the evolutionarily distant *E. coli* and *B. subtilis*.

## Role of the Stringent Response in Chromosome Replication

In *E. coli*, chromosome replication initiates at a single origin of replication, *oriC*, which contains an AT-rich region and multiple binding-sites for the initiator protein, DnaA (Leonard and Mechali, 2013). DnaA belongs to the family of AAA + proteins and binds ATP and ADP with similar affinity (Sekimizu et al., 1987), of which only the ATP-bound form, DnaA^ATP^, is required for oligomerization at *oriC*, and hence active for initiation (reviewed in Skarstad and Katayama, 2013; Riber et al., 2016). Origin unwinding leads to loading of DNA helicase, DnaB, onto single-stranded DNA (ssDNA) by the helicase loader, DnaC, followed by recruitment of primase, DnaG, as well as assembly of two replisomes to direct replication bidirectionally, until the replication forks meet and terminate at the terminus region, opposite to *oriC* (Kornberg and Baker, 1992). In *B. subtilis*, chromosome replication is mediated by the same overall steps, but the bipartite replication origin, containing two DnaA-box clusters separated by the *dnaA* gene (Moriya et al., 1992), is structurally different as compared to the continuous replication origin of *E. coli*. Also, assembly of the helicase, DnaC, onto ssDNA by the helicase loader, DnaI, occurs via a different mechanism known as “ring assembly” (Soultanas, 2012), but the following recruitment of DnaG primase and assembly of the replication elongation machinery is largely similar to that of *E. coli* (reviewed by Jameson and Wilkinson, 2017).

Highly different strategies have been adopted for (p)ppGpp-mediated chromosome replication inhibition in *E. coli* and *B. subtilis*. It is widely accepted that replication arrest in *B. subtilis* occurs downstream from the origin (i.e., on the elongation level), whereas replication inhibition in *E. coli* occurs at the initiation level, suggesting that stringent cell cycle arrest points differ between *E. coli* and *B. subtilis* (Levine et al., 1991).

### (p)ppGpp-Mediated Inhibition of Initiation of Chromosome Replication

High levels of (p)ppGpp inhibit chromosome replication initiation in *E. coli* (Levine et al., 1991; Schreiber et al., 1995; Ferullo and Lovett, 2008; Riber and Lobner-Olesen, 2020), but the exact mechanism responsible for this inhibition has been somewhat unclear. However, several recent papers have made crucial discoveries adding valuable insight into this area of research.

Previously, the transcriptional activity of both *dnaA* operon promoters was reported to be stringently controlled (Chiaramello and Zyskind, 1990; Zyskind and Smith, 1992), suggesting that reduced *dnaA* gene transcription, and hence lowered *de novo* DnaA protein synthesis, could explain the initiation arrest observed in the presence of elevated (p)ppGpp levels. This was supported by a recent study, reporting that continued DnaA synthesis, expressed from a (p)ppGpp-insensitive T7 RNAP-dependent promoter, allowed for replication initiation during (p)ppGpp accumulation (Riber and Lobner-Olesen, 2020). Additionally, it was reported that polyphosphate during the stringent response activates Lon protease to degrade DnaA^ADP^. As several regulatory systems work in concert to convert DnaA^ATP^ into DnaA^ADP^ (Katayama et al., 1998; Kato and Katayama, 2001; Kasho and Katayama, 2013), this indirectly lowers the amount of active DnaA^ATP^, causing replication initiation to cease (Gross and Konieczny, 2020). However, degradation of DnaA has been reported only for *Caulobacter crescentus*, and not for *E. coli* (Gorbatyuk and Marczynski, 2005; Katayama et al., 2010). Also, recent data give no indication of DnaA degradation during (p)ppGpp accumulation (Riber and Lobner-Olesen, 2020).

Interestingly, several studies address the importance of DnaA activity, i.e., the DnaA^ATP^-to-DnaA^ADP^ ratio, during (p)ppGpp accumulation. Continuous *de novo* DnaA synthesis was found to allow for new rounds of replication initiation during (p)ppGpp accumulation (Riber and Lobner-Olesen, 2020). As the level of ATP is more abundant than ADP in the cell (Petersen and Møller, 2000), and because DnaA binds these nucleotides with similar affinity (Sekimizu et al., 1987), *de novo* synthesized DnaA will be mainly ATP-bound, which ensures that the pool of DnaA^ATP^ is continuously being replenished. Thus, while overall cell growth ceases due to (p)ppGpp accumulation DnaA^ATP^ continues to increase due to *de novo* synthesis, which in turn allows for continued replication initiation during high levels of (p)ppGpp. In contrast, overproduction of DnaA during otherwise normal cell growth does not notably increase the DnaA^ATP^ level (Flatten et al., 2015). Following induction of (p)ppGpp in such cells, transcription of *dnaA* will be repressed, which results in insufficient accumulation of active DnaA^ATP^ to sustain further initiations (Kraemer et al., 2019).

Altogether, these observations suggest that (p)ppGpp-mediated replication initiation inhibition occurs through prevention of *de novo* DnaA synthesis, which lowers both the amount and activity (i.e., ATP-bound status) of DnaA. In agreement with this, (p)ppGpp fails to arrest replication initiation in cells where a hyperactive DnaA protein, mimicking ATP-bound DnaA, is overproduced (Kraemer et al., 2019).

Limitation of DnaA does, however, not seem to be the sole mechanism responsible of (p)ppGpp-mediated replication initiation inhibition. Recent studies emphasize lack of transcriptional activation of *oriC* to explain the negative effect of (p)ppGpp on initiation. Here, (p)ppGpp-driven reduction in transcriptional activity of promoters located close to *oriC*, presumably preventing introduction of negative supercoils in the wake of the migrating RNA polymerase complex, was suggested to cause less transcriptional activation of the origin, hence inhibiting initiation (Kraemer et al., 2019). Also, DNA gyrase (*gyrA*) and topoisomerase IV (*parC*) expression was found to be inhibited by high levels of (p)ppGpp, and the negative superhelicity of *oriC* was suggested to be lowered, despite not actually being measured (Fernandez-Coll et al., 2020).

Both *mioC* and *gidA* promoters, located adjacent to *oriC*, can be deleted without measurable effects (Lobner-Olesen and Boye, 1992; Bates et al., 1997; Lies et al., 2015), showing that they are dispensable for replication initiation during normal growth. However, when *oriC* becomes sufficiently impaired for initiation, such as when DnaA box R4 is deleted, transcription from these promoters becomes important (Bates et al., 1997). This is supported by the initiation kinetics of rifampicin and chloramphenicol. As rifampicin inhibits transcription initiation (Hartmann et al., 1967) rifampicin-treated cells will gradually stop to accumulate DnaA, but translation will continue as long as intact *dnaA* mRNA is present. On the other hand, chloramphenicol treatment will immediately block DnaA translation (Vazquez, 1979). Yet, chloramphenicol did not inhibit initiation as fast as rifampicin (Lark, 1972; Messer, 1972; Riber and Lobner-Olesen, 2020). As transcription is still on-going in chloramphenicol treated cells, this supports the ability of transcriptional activation of *oriC* to allow for extra initiations during suboptimal, e.g., DnaA limiting, conditions.

**FIGURE 1:**
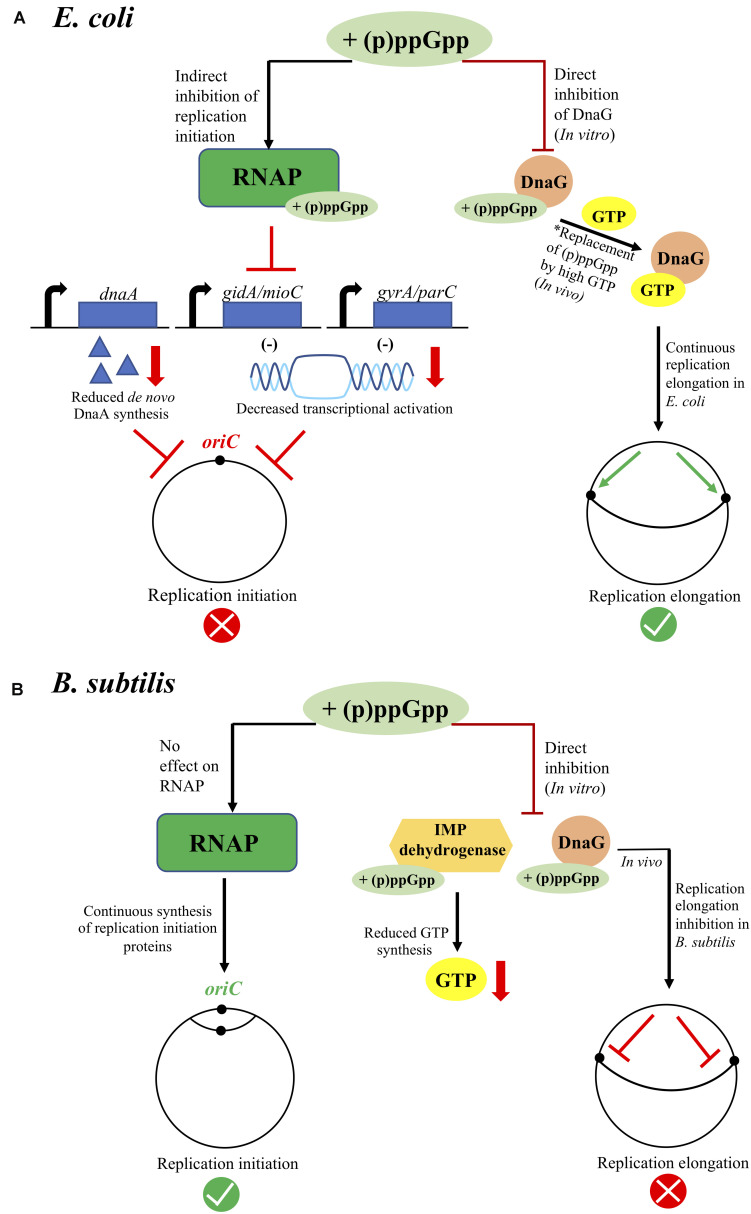
Overview of (p)ppGpp-meditated inhibition of chromosome replication in *E. coli* **(A)** and *B. subtilis* **(B)**. In *E. coli* **(A)** replication inhibition occurs at the initiation level during (p)ppGpp accumulation. Here, (p)ppGpp binds the RNA Polymerase (RNAP), which indirectly affects the global gene expression profile through RNAP-driven transcriptional reprogramming. Downregulated gene transcripts include *dnaA, gidA, mioC, gyrA*, and *parC*, leading to lack of *de novo* DnaA synthesis and possibly lowered transcriptional activation of *oriC*, which all together contribute in arresting replication initiation during (p)ppGpp accumulation. Also, (p)ppGpp binds DnaG primase *in vitro*, but replication elongation remains unaffected *in vivo*. As GTP levels are not significantly reduced in *E. coli* during (p)ppGpp accumulation, and since GTP also binds DnaG, we hypothesize that GTP might outcompete (p)ppGpp in binding DnaG *in vivo* (this hypothesis is marked as *). In *B. subtilis* **(B)** replication inhibition occurs at the elongation level during (p)ppGpp accumulation. Here, (p)ppGpp binds IMP dehydrogenase, lowering the pool of available GTP, as well as DnaG. The significantly reduced level of GTP leads to DnaG being susceptible to strongly binding (p)ppGpp *in vivo*. Substantial replication occurs at the *B. subtilis* origin during (p)ppGpp accumulation, possibly because (p)ppGpp does not directly bind RNAP, excluding any RNAP-driven transcriptional reprogramming, or any replication initiation proteins.

In conclusion, failure to *de novo* synthesize DnaA (i.e., reduced *dnaA* transcription) and to replenish the DnaA^ATP^ pool along with lowered transcriptional activation of *oriC* (i.e., reduced *gidA/mioC* and/or *gyrA/parC* transcription) contribute in arresting replication initiation during (p)ppGpp accumulation in *E. coli* (Figure 1A; left). However, it is difficult to quantitate the exact contribution from each of those mechanisms.

### (p)ppGpp-Mediated Inhibition of Elongation of Chromosome Replication

In contrast to *E. coli*, substantial replication occurs at the *B. subtilis* origin following induction of the stringent response. Also, regulation of chromosome replication initiation was shown to be independent of (p)ppGpp accumulation in *B. subtilis* (Levine et al., 1991; Murray and Koh, 2014). This indicates that (p)ppGpp might not regulate the synthesis of replication initiation proteins and/or transcriptional activation of *oriC* in *B. subtilis.* The lack of RNAP-driven transcriptional reprogramming due to *B. subtilis* RNAP not being a direct target of (p)ppGpp partly supports the latter (Figure 1B; left). Replication was instead shown to be arrested at distinct termination sites located approximately 200 kb downstream on either side of *oriC* (Levine et al., 1991), suggesting (p)ppGpp-mediated inhibition of chromosome replication in *B. subtilis* to be regulated at the post-initiation level.

By using genomic microarrays to monitor the progression of replication forks in synchronized cell cultures of *B. subtilis*, it was later revealed that starvation-induced replication arrest occurred throughout the chromosome, irrespective of the location of the replication forks. A direct (p)ppGpp-mediated inhibition of DNA primase (DnaG) activity, known to affect replication fork progression (Wu et al., 1992; Lee et al., 2006), was proposed to underlie the observed replication elongation arrest (Wang et al., 2007). This inhibition was found to be dose-dependent, suggesting that the severity of stress (i.e., concentration of (p)ppGpp) is tightly coupled to an equivalent reduction in replication progression rate, thus providing a tunable stress response (Wang et al., 2007; Denapoli et al., 2013). Interestingly, replication forks arrested in the presence of high levels of (p)ppGpp did not recruit the SOS response protein RecA, indicating that stalled forks were not disrupted, but reversibly halted with the ability to restart replication upon nutrient availability (Wang et al., 2007). These observations support that (p)ppGpp-mediated primase inhibition serves to maintain genome integrity during periods of stress.

Another factor that might contribute to the strong (p)ppGpp inhibition of progressing replication forks in *B. subtilis* is the equivalent decrease in the cellular pool of GTP available for continued DNA strand extension. This decrease is caused by increased consumption of GTP during (p)ppGpp biosynthesis, and by a direct inhibition of the activity of inosine monophosphate (IMP) dehydrogenase that catalyzes an early step in GTP biosynthesis (Lopez et al., 1981; Figure 1B; right).

(p)ppGpp binds and inhibits the *E. coli* DnaG primase *in vitro* (Maciag et al., 2010; Rymer et al., 2012). To date, no other replication proteins in *E. coli*, including DnaA, have been reported as direct targets for (p)ppGpp (Zhang et al., 2018; Wang et al., 2019). Obviously, this finding contradicts decades of research stating that ongoing rounds of replication are continued until completion following induction of the stringent response in *E. coli*, proposing that DNA replication elongation is not arrested during (p)ppGpp accumulation *in vivo* (Schreiber et al., 1995; Ferullo and Lovett, 2008; Kraemer et al., 2019; Riber and Lobner-Olesen, 2020). DeNapoli et al. did quantify genome-wide replication fork progression in *E. coli* and revealed that the replication elongation rate was modestly reduced by (p)ppGpp induction, but possibly the response was restricted to acute stress conditions (Denapoli et al., 2013).

Factors preventing binding of (p)ppGpp to DnaG, or the competing action between RNAP and DnaG in binding (p)ppGpp, were suggested to explain the lack of effect on DnaG activity *in vivo* (Maciag et al., 2010). Indeed, (p)ppGpp was found to bind DnaG at partially overlapping sites with nucleotides and inhibit primase activity in a GTP-concentration dependent manner (Rymer et al., 2012). As GTP levels are not reduced by more than 50% in *E. coli* during the stringent response (Varik et al., 2017), whereas *B. subtilis* experiences a significant drop in GTP concomitant with (p)ppGpp accumulation (Ochi et al., 1982), this supports a stronger (p)ppGpp-mediated binding to- and inhibition of DnaG in *B. subtilis*, hence leading to a more potent inhibition of replication elongation as compared to *E. coli* (Figures 1A,B; right).

## Role of the Stringent Response in DNA Damage and Repair

Bacterial genomic integrity is often threatened by DNA damage induced either by natural fork breakage, fork stalling, replication-transcription collision, or by external threats such as radiation and DNA modifying drugs (Kuzminov, 1999). Faithful damage repair orchestrated by DNA repair proteins is essential to maintain genomic integrity, chromosomal replication and cell viability. Accordingly, mutants lacking repair proteins are sensitive to DNA damaging agents and are less viable (Van Houten, 1990; Kuzminov, 1999; Sinha et al., 2020). Since (p)ppGpp binding to RNAP in *E. coli* destabilizes the open promoter complexes, it is expected to modulate replication-transcription collision and to play a role in maintaining genomic integrity.

The observation that loss of both RelA and SpoT (ppGpp^0^ strain), i.e., inability to synthesize (p)ppGpp, enhanced UV sensitivity of an *E. coli ruvAB* mutant, suggested a possible role of (p)ppGpp in facilitating DNA repair (McGlynn and Lloyd, 2000). RuvAB along with RuvC play a role in branch migration and resolution of Holliday junctions, formed during RecBCD-RecA-mediated DNA double-strand break (DSB) repair and RecFOR-RecA-mediated gap repair (Kuzminov, 1999; Sinha et al., 2020). Interestingly, a slight increase in the basal level of (p)ppGpp by using the *spoT1* allele, having reduced (p)ppGpp hydrolytic activity, improved UV survival of the *ruvAB* mutant (McGlynn and Lloyd, 2000). Thus, high (p)ppGpp increases/promotes viability, whereas no (p)ppGpp increases UV sensitivity of the *ruvAB* mutant. The ppGpp^0^ strain alone was also found to be UV sensitive (McGlynn and Lloyd, 2000).

The ppGpp^0^ strain displays an amino acid auxotrophy phenotype and accumulates suppressor mutations (known as “stringent mutants”) that allow cells to grow in minimal medium lacking amino acids. These suppressor mutations occur in RNAP subunits encoded by *rpoB* and *rpoC* (Zhou and Jin, 1998; McGlynn and Lloyd, 2000), and were shown to destabilize the transcriptional complex in a manner similar to (p)ppGpp binding to RNAP (Trautinger et al., 2005). Remarkably, some of these suppressor mutations (denoted *rpo*^∗^) significantly improved survival of the Δ*relA* Δ*spoT* Δ*ruvAB* strain after UV treatment (McGlynn and Lloyd, 2000).

**FIGURE 2:**
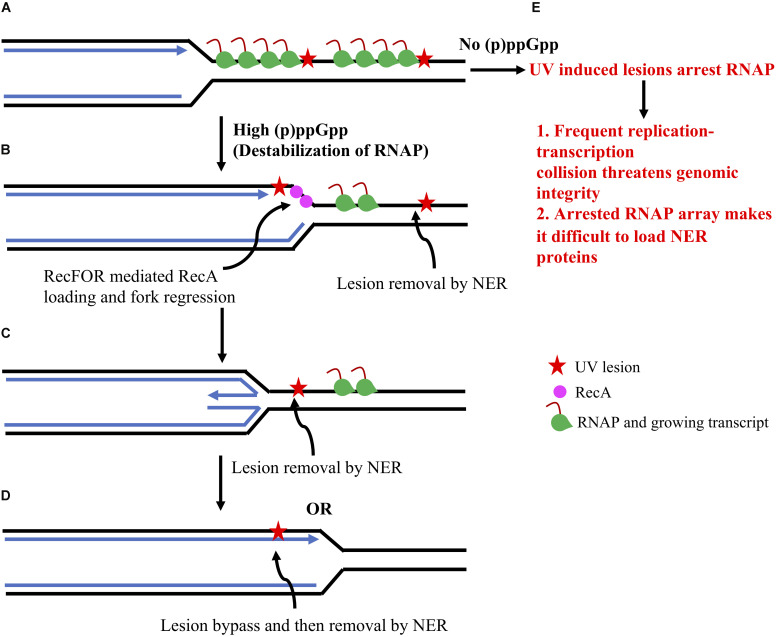
Overview of (p)ppGpp-meditated repair of UV induced DNA damage in *E. coli*. The UV induced DNA lesions arrest RNAP and halt transcription progression **(A)**. This can lead to frequent replication-transcription collision. In **(A)**, only co-directional collision has been shown but there is an equal possibility for head-on collision and both threaten genomic integrity. This scenario will probably be escalated in absence of (p)ppGpp since the RNAP array will be stably arrested for a long time in absence of (p)ppGpp **(A–E)**. Whereas, (p)ppGpp binding to RNAP will destabilize it and remove it from the DNA template. Removal of RNAP will help in two ways: 1. It will create space to load NER proteins and remove/repair DNA lesions. 2. It will help the replication fork to progress toward DNA lesions **(B)**. Arrested replication forks can get reversed with the help of RecFOR mediated RecA loading and fork regression **(C)**. DNA synthesis and resetting of the replication fork will help in lesion bypass **(D)**. DNA lesions can be removed and repaired by NER pathways either at the **(C,D)** step. This model is adapted from Trautinger et al. (2005).

Thus, it was proposed that (p)ppGpp/*rpo*^∗^-mediated destabilization of transcriptional complexes reduces the occurrence of stalled RNAP on DNA, hence allowing free space for efficient excision repair of UV-induced DNA lesions and for simultaneous facilitation of replication fork progression by avoiding replication-transcription conflicts (McGlynn and Lloyd, 2000; Trautinger and Lloyd, 2002; Trautinger et al., 2005). Additionally, it was shown that (p)ppGpp-mediated suppression of *ruvAB* mutant UV sensitivity is complex and requires RecA, RecG, and PriA, but not RecBCD, and was proposed to involve replication fork stalling, regression and restart (McGlynn and Lloyd, 2000). Since replication fork stalling, regression and restart are the major reactions following UV irradiation in *E. coli* cells (Khan and Kuzminov, 2012), the most plausible explanation for the UV resistance phenotype of *spoT1 ruvAB* (or *rpo*^∗^ *ruvAB*) cells would be destabilization of the RNAP array allowing replication forks to directly encounter DNA lesions followed by an active fork regression and lesion bypass, instead of fork breakage, to facilitate replication restart (Trautinger et al., 2005; Figure 2).

In contrast to UV, high (p)ppGpp (or *rpo*^∗^) cannot suppress sensitivity of the Δ*ruvAB* strain against exposure to mitomycin C (MMC) or γ rays (McGlynn and Lloyd, 2000). It should be noted that DNA lesions generated by both UV and MMC are removed/repaired by nucleotide excision repair (NER) (Van Houten, 1990). However, MMC treatment generates inter-strand crosslinks that most often get converted into DSBs, whereas UV treatment induces intra-strand pyrimidine dimers with generation of DSBs being primarily dependent on replication fork stalling at the lesion site (Khan and Kuzminov, 2012). These observations exclude a direct role of (p)ppGpp in DSBs repair.

### Transcription-Coupled DNA Repair (TCR)

Another study, corroborating the above finding, confirmed that *E. coli* ppGpp^0^ cells were highly sensitive to UV radiation, 4-nitroquinoline-1-oxide (4NQO), and nitrofurazone (NFZ) (Kamarthapu et al., 2016). These agents induce formation of DNA adducts, which are mainly removed and repaired by NER pathways (Ikenaga et al., 1975; Ona et al., 2009). Remarkably, wild-type cells rapidly accumulated a 20-fold increase in (p)ppGpp when treated with 4NQO or NFZ, suggesting that DNA lesions induce (p)ppGpp synthesis. However, the mechanism of (p)ppGpp synthesis during these treatments remains to be determined (Kamarthapu et al., 2016).

TCR is defined by an active transcription-dependent increase in excision repair of lesions on the transcribed DNA strand in comparison to the non-transcribed strand (Mellon and Hanawalt, 1989). Two factors, Mfd and UvrD, promote TCR by two different pathways: by pushing RNAP forward of the DNA lesion and by promoting RNAP backtracking, respectively, followed by recruitment of NER proteins, such as UvrAB at the lesion site (Mellon and Hanawalt, 1989; Kamarthapu and Nudler, 2015). Interestingly, the preference for repairing the transcribed strand rather than the non-transcribed strand was abolished in ppGpp^0^ cells suggesting that (p)ppGpp is crucial for TCR. Since the sensitivity of ppGpp^0^ cells to UV, 4NQO or NFZ was epistatic to *uvrD* mutant sensitivity, it was proposed that (p)ppGpp potentiates the pro-backtracking activity of UvrD (Kamarthapu et al., 2016). The role of (p)ppGpp in facilitating TCR can also occur independent of UvrD either by promoting RNAP backtracking by destabilizing and removing RNAP complexes from tightly packed arrays at the highly transcribed ribosomal genes, thus creating space for backtracking, or by reducing the number of ribosomes trailing RNAP to make space for backtracking (Rasouly et al., 2017). However, extensive backtracked RNAP might increase the risk of replication-transcription collision and has the capacity to induce DSBs and genomic instability (Dutta et al., 2011). The conundrum is perhaps resolved by (p)ppGpp-mediated inhibition of replication initiation, thus minimizing the frequency of replication-transcription collisions when RNAP backtracking is needed to repair genotoxic lesions on DNA.

In *B. subtilis*, the SMC-ScpAB complex is important for chromosome condensation and segregation, and Δ*smc* mutants exhibit pleiotropic phenotypes including defects in chromosome condensation, segregation, DNA repair and viability at high temperature. Upregulation of the stringent response has been shown to suppress chromosome segregation defects, hypersensitivity to gyrase inhibitors and restore viability of Δ*smc* mutants (Benoist et al., 2015). Since the stringent response slows down replication elongation in *B. subtilis*, it might be possible that slow replication allows chromosome segregation to occur even in the absence of the SMC-ScpAB complex. This hypothesis finds support as Δ*smc* mutant cells grow well in minimal medium (i.e., slow growth conditions) as compared to no growth in rich medium (i.e., fast growth conditions) at 37°C (Benoist et al., 2015). Similar studies for the role of the stringent response in chromosome segregation mutant cells of *E. coli* have not been reported.

## Concluding Remarks

Based on the highlights presented throughout this review, the stringent response has clearly proven to affect both bacterial chromosome replication and DNA repair activities. However, whereas (p)ppGpp accumulation negatively affects replication initiation and replication elongation in *E. coli* and *B. subtilis*, respectively, the effect of (p)ppGpp-mediated modulation of DNA repair activities seems positive. Indeed, the absence of (p)ppGpp makes *E. coli* cells sensitive to UV and other DNA damaging agents, and studies suggest a role of (p)ppGpp in enhancing the efficiency of NER-dependent repair of DNA lesions, most likely by destabilizing RNAP complexes and making space for recruitment of NER proteins. Interestingly, these observations might be coupled to (p)ppGpp-mediated replication inhibition, which prevents replication-transcription collisions and/or reduces the frequency of replication forks meeting the UV lesions, thus assisting efficient NER-mediated repair. This intriguing hypothesis, connecting the negative effect of (p)ppGpp on replication to (p)ppGpp-driven stimulation of DNA repair activity, can easily be tested by using a system where (p)ppGpp-dependent replication inhibition is abrogated as recently described (Riber and Lobner-Olesen, 2020).

## Author Contributions

AS, AL-O, and LR wrote the manuscript. AS and LR designed and prepared the figures. All authors contributed to the article and approved the submitted version.

## Conflict of Interest

The authors declare that the research was conducted in the absence of any commercial or financial relationships that could be construed as a potential conflict of interest.
